# Supplementary material for: Skin Microbiota, Immune Cell, and Skin Fibrosis: A Comprehensive Mendelian Randomization Study
Source: Biomedicines. 2024 Oct 21;12(10):2409. doi: 10.3390/biomedicines12102409 (PMC11505207; doi:10.3390/biomedicines12102409)
Supplement: Supplementary file 1 [file biomedicines-12-02409-s001.zip › Supplementary Figure S1-S4.pdf]

Supplementary Figures

Figure S1. MR leave-one-out sensitivity analysis for skin microbiota on skin fibrosis.

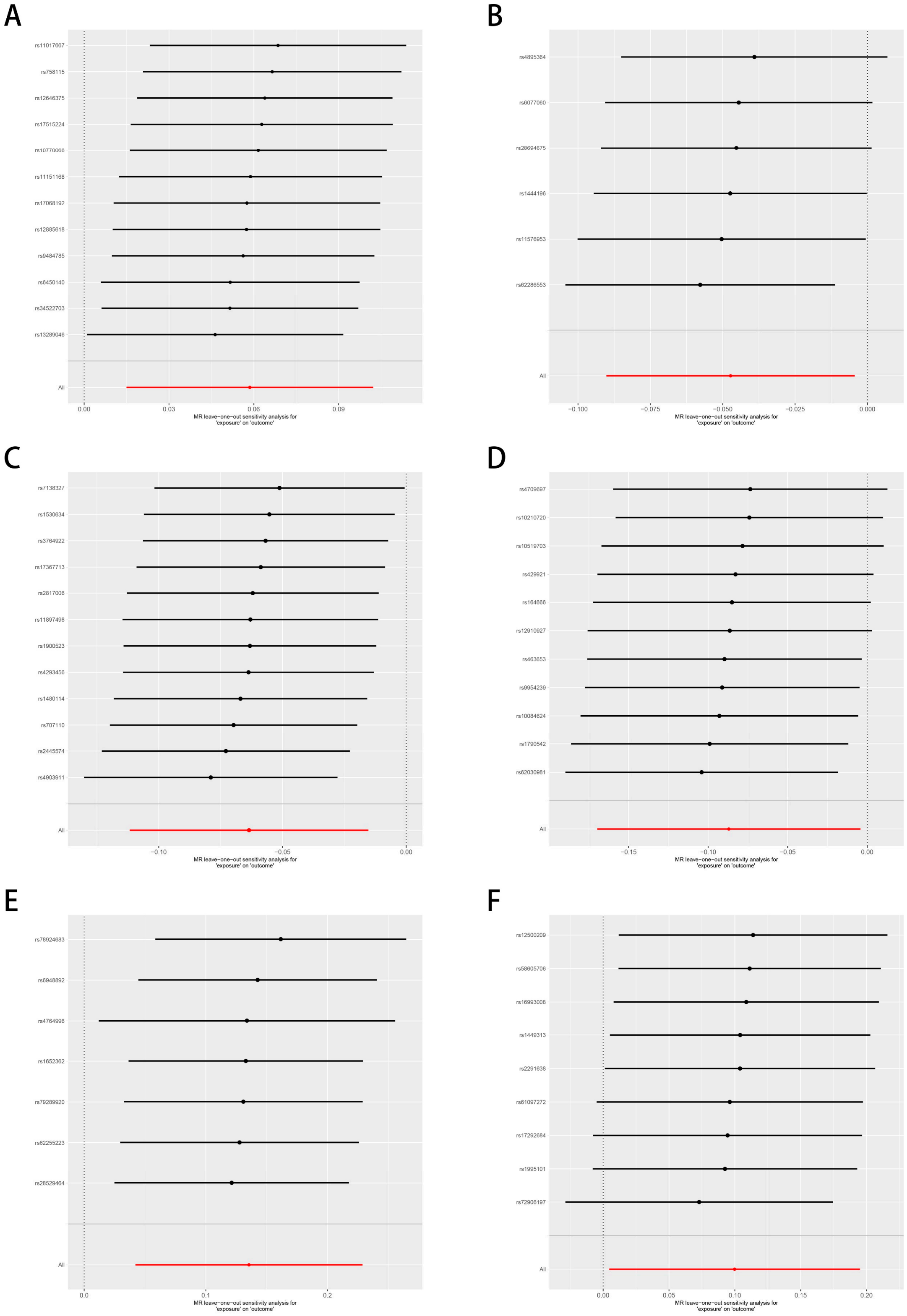

Figure S1: MR leave-one-out sensitivity analysis for skin microbiota on skin fibrosis. **A**. MR leave-one-out sensitivity analysis for *Enhydrobacter* (unc.) (moist skin) on hypertrophic scar. **B**. MR leave-one-out sensitivity analysis for *Anaerococcus* (unc.) (dry skin) on *Hypertrophic scar* trait. **C**. MR leave-one-out sensitivity analysis for *Class: betaproteobacteria* (moist skin) on *Hypertrophic scar* trait. **D**. MR leave-one-out sensitivity analysis for *S. epidermidis* (moist skin) on localized scleroderma. **E**. MR leave-one-out sensitivity analysis for *S. epidermidis* (dry skin) on localized scleroderma. **F**. MR leave-one-out sensitivity analysis for *R. mucilaginosa* (dry skin) on localized scleroderma.

Figure S2. Forest plots for casual effects of skin microbiota on skin fibrosis.

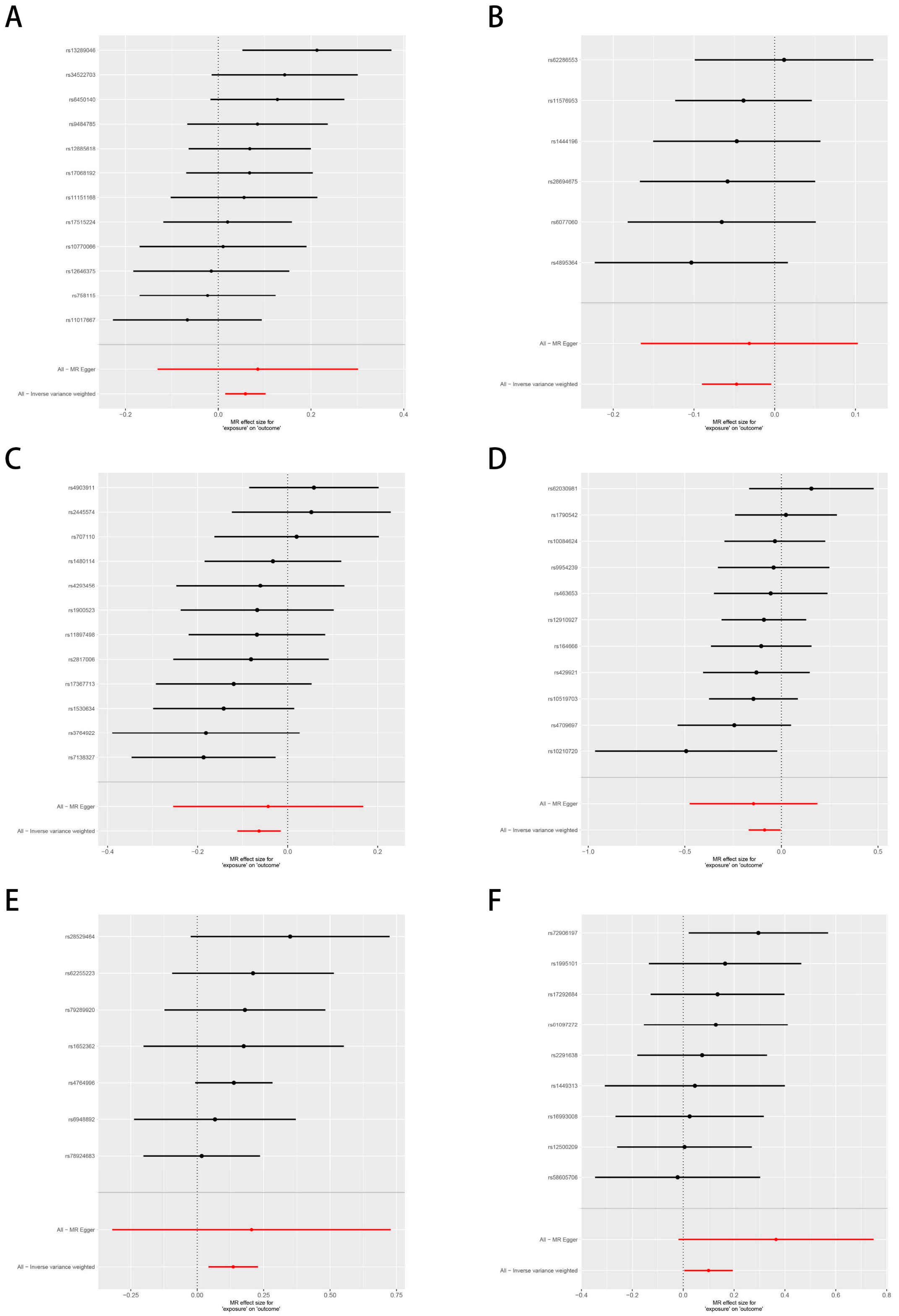

Figure S2: Forest plots for causal effects of skin microbiota on skin fibrosis. **A.** The forest plot for a causal effect of *Enhydrobacter* (unc.) (moist skin) on *Hypertrophic scar* trait. **B.** The forest plot for a causal effect of *Anaerococcus* (unc.) (dry skin) on *Hypertrophic scar* trait. **C.** The forest plot for a causal effect of *Class: betaproteobacteria* (moist skin) on *Hypertrophic scar* trait. **D.** The forest plot for a causal effect of *S. epidermidis* (moist skin) on *Localized-scleroderma* trait. **E.** The forest plot for a causal effect of *S. epidermidis* (dry skin) on *Localized-scleroderma* trait. **F.** The forest plot for a causal effect of *R. mucilaginosa* (dry skin) on *Localized-scleroderma* trait.

Figure S3. Scatter plots for casual effects of skin microbiota on skin fibrosis.

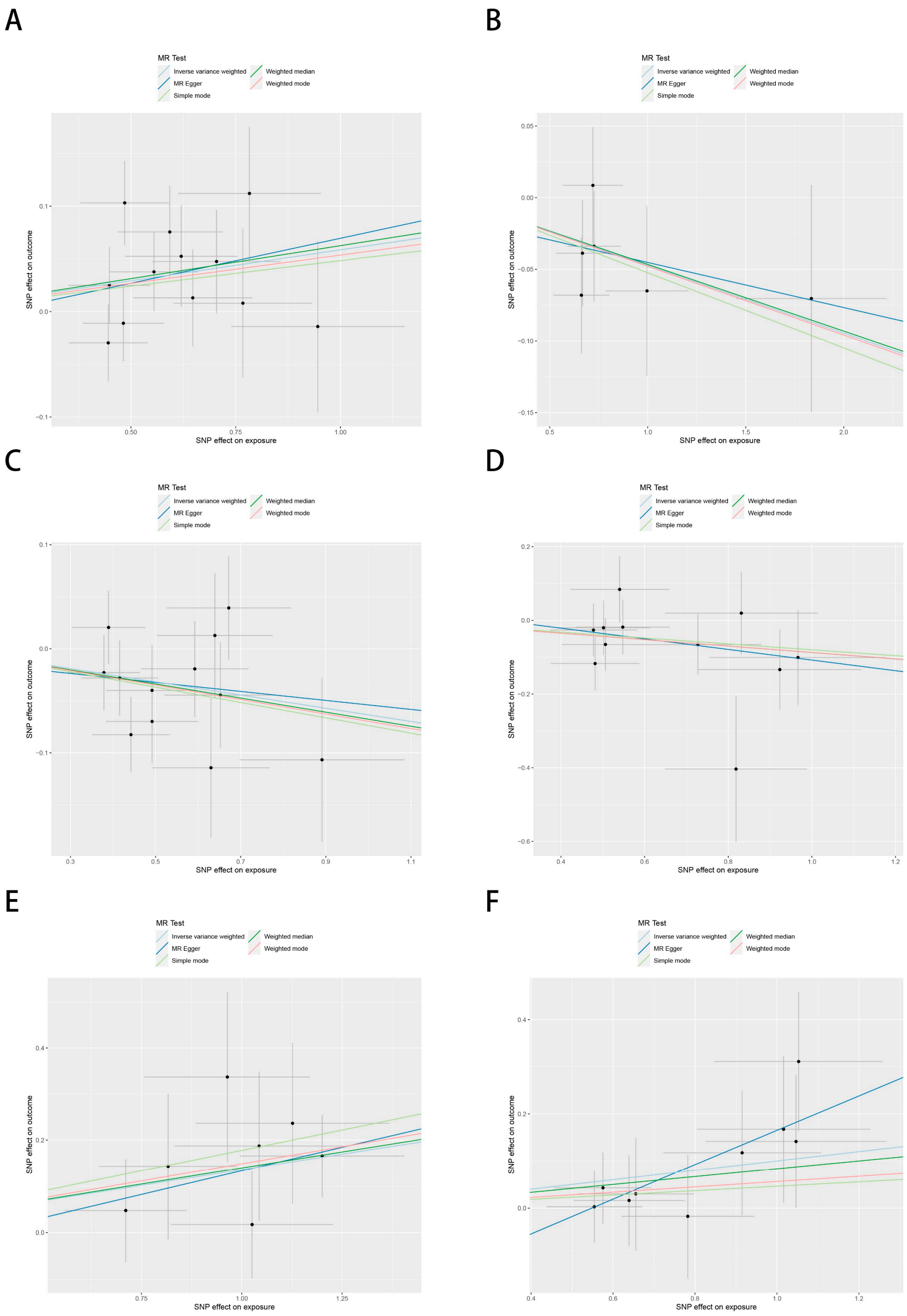

Figure S3: Scatter plots for causal effects of skin microbiota on skin fibrosis. **A.** The scatter plot for a causal effect of *Enhydrobacter* (unc.) (moist skin) on *Hypertrophic scar* trait. **B.** The scatter plot for a causal effect of *Anaerococcus* (unc.) (dry skin) on *Hypertrophic scar* trait. **C.** The scatter plot for a causal effect of *Class: betaproteobacteria* (moist skin) on *Hypertrophic scar* trait. **D.** The scatter plot for a causal effect of *S. epidermidis* (moist skin) on *Localized-scleroderma* trait. **E.** The scatter plot for a causal effect of *S. epidermidis* (dry skin) on *Localized-scleroderma* trait. **F.** The scatter plot for causal effect of *R. mucilaginosa* (dry skin) on *Localized-scleroderma* trait.

Figure S4. Funnel plots for causal effects of skin microbiota on skin fibrosis.

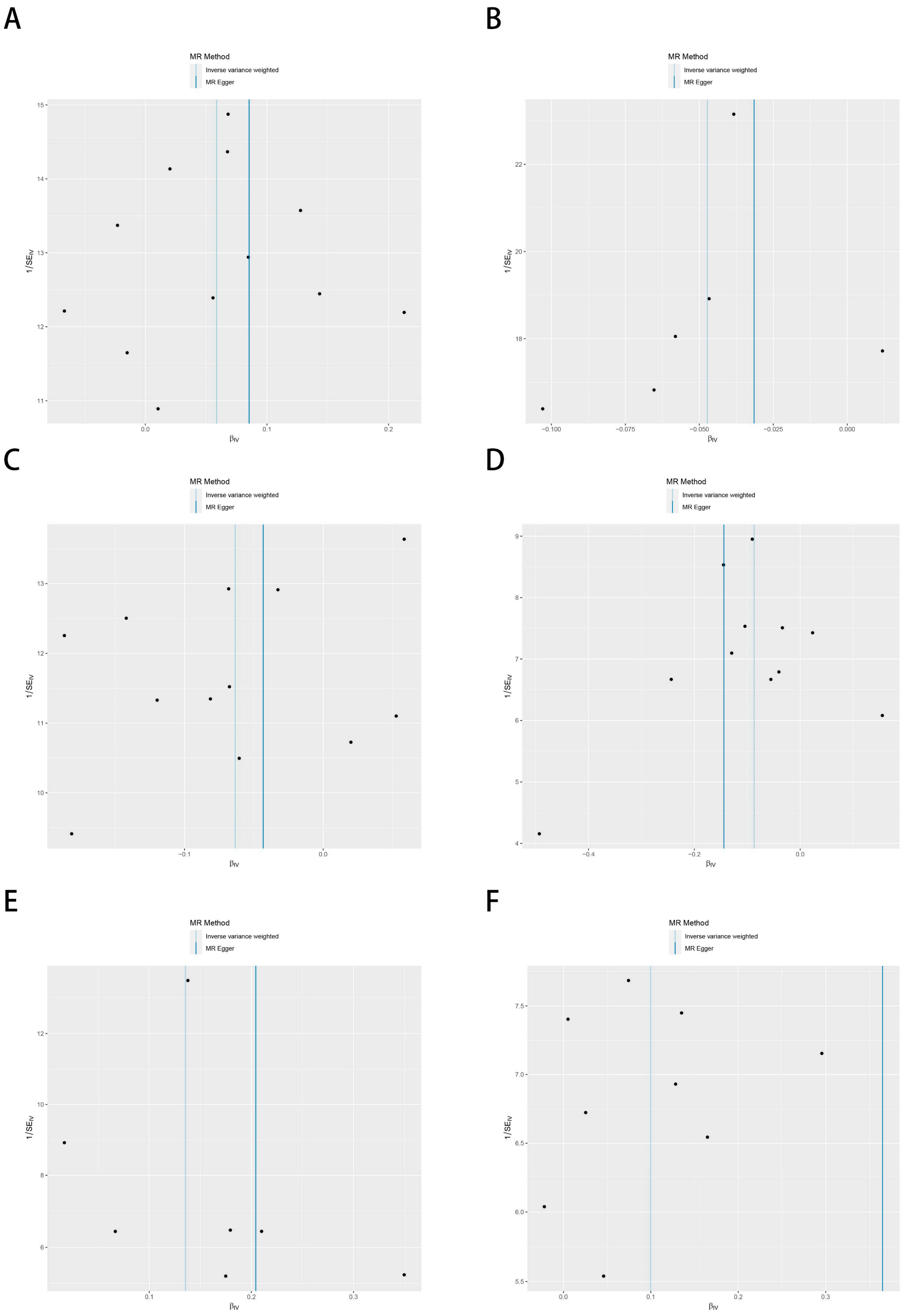

Figure S4: Funnel plots for causal effects of skin microbiota on skin fibrosis. **A.** The funnel plot for a causal effect of *Enhydrobacter* (unc.) (moist skin) on *Hypertrophic scar* trait. **B.** The funnel plot for a causal effect of *Anaerococcus* (unc.) (dry skin) on *Hypertrophic scar* trait. **C.** The funnel plot for a causal effect of *Class: betaproteobacteria* (moist skin) on *Hypertrophic scar* trait. **D.** The funnel plot for a causal effect of *S. epidermidis* (moist skin) on *Localized-scleroderma* trait. **E.** The funnel plot for a causal effect of *S. epidermidis* (dry skin) on *Localized-scleroderma* trait. **F.** The funnel plot for a causal effect of *R. mucilaginosa* (dry skin) on *Localized-scleroderma* trait.
